# Sensorimotor recalibration of postural control strategies occurs after whole body vibration

**DOI:** 10.1038/s41598-022-27117-7

**Published:** 2023-01-10

**Authors:** Isotta Rigoni, Giulio Degano, Mahmoud Hassan, Antonio Fratini

**Affiliations:** 1 Biomedical Engineering, College of Engineering and Physical Sciences, Aston University, Birmingham, UK; 2 EEG and Epilepsy Unit, Clinical Neuroscience Department, University Hospital and Faculty of Medicine of Geneva, Geneva, Switzerland; 3 University of Geneva, Geneva, Switzerland; 4 Department of Engineering, School of Technology, Reykjavík University, Reykjavík, Iceland

**Keywords:** Biomedical engineering, Neurophysiology

## Abstract

Efficient postural control results from an effective interplay between sensory feedbacks integration and muscle modulation and can be affected by ageing and neuromuscular injuries. With this study, we investigated the effect of whole-body vibratory stimulation on postural control strategies employed to maintain an upright posture. We explored both physiological and posturography metrics, through corticomuscular and intramuscular coherence, and muscle networks analyses. The stimulation disrupts balance in the short term, but leads to a greater contribution of cortical activity, necessary to modulate muscle activation via the formation of (new) synergies. We also observed a reconfiguration of muscle recruitment patterns that returned to pre-stimulation levels after few minutes, accompanied by a slight improvement of balance in the anterior–posterior direction. Our results suggest that, in the context of postural control, appropriate mechanical stimulation is capable of triggering a recalibration of the sensorimotor set and might offer new perspectives for motor re-education.

## Introduction

For a few decades, it has been assumed that balance was achieved by merely passive local reflexes, as mammals were found to be able to stand still solely by tonic muscle contractions^1^. This view has been recently challenged and a new working hypothesis has been proposed suggesting that maintaining balance—as well as any other human movement—requires active planning, and therefore involves global multisensory integration and processing of a higher level^2,3^. According to this perspective, as we stand, inputs from the external environment are processed online by the central nervous system (CNS) to maintain a stable upright stance^4^. Vestibular, visual and somatosensory cues are integrated to regulate muscle contraction over time, with the ultimate goal of adjusting the centre of mass position, preventing falls^4^. Efficient postural control results from an effective interplay between sensory feedback integration and cortical drive to the muscles, which allows to *detect*, *react to* and *correct* the perturbations that normally occur while standing upright^5,6^. Two effective tools are available to investigate such communication occurring between muscles and the brain: intermuscular coherence (IMC) and corticomuscular coherence (CMC)^7^.

IMC is thought to represent the common neural input that, originating from efferent and afferent pathways, is fed to motor units (MUs) of different muscles^8^. To facilitate motor control, the CNS is thought to deal with the several degrees of freedom of the musculoskeletal system by *coordinating* muscle activation, i.e. recruiting muscles in groups^9^. The state-of-the-art approach to IMC analyses is to model muscle co-activation via complex network analysis, which has largely been employed to understand the organisation of cortical activity^10^. Studying muscle-networks across tasks revealed the synchronous modulation of muscles to be functional, showing the potential of such analyses to uncover mechanisms of interest^11^.

Similarly, CMC has been historically thought to represent signalling from pyramidal neurons to spinal motor neurons, which subsequently control the corresponding muscle fibres^12^. Recent findings nevertheless suggest that CMC also reflects afferent couplings, i.e., the ascending flow of information that from muscle spindles reaches somatosensory areas in the brain^13^. This is further supported by the detection of oscillations in beta band (15–30 Hz) both in the motor (M1) and somatosensory (S1) cortex^14^, that are coherent with the activity recorded from contralateral contracting muscles^15^. The presence of activity in beta frequency range has been vastly documented in the human motor system and it is the reason why CMC is mostly investigated in beta band^13,16^. Studies looking at CMC are frequently designed to involve steady-state motor output, such as precision grip and isometric contractions^17–22^, or dynamic voluntary movements^23–26^, such as walking. Very few studies evaluated CMC during standing balance^27–29^ and even less highlighted any CMC during postural control^30–32^.

It is however sensible to investigate postural control mechanisms by inspecting couplings in such frequency range. Oscillations between 15 and 30 Hz have in fact been linked to the attempt of preserving the ongoing sensorimotor state. More in detail, Engel and Fries suggest that beta band activity (BBA) acts as a facilitator of proprioceptive feedback processing^33^. In the intended attempt of preserving the status quo and regaining balance after a disturbance, the enhanced activity observed in beta band is thought to promote the handling of proprioceptive signals coming from the periphery, on which we rely for the perception and recalibration of the sensorimotor system^34^.

Among all the inputs that we use online to modify our posture, the somatosensory ones play perhaps the most important role: since proprioceptive thresholds are smaller than visual and vestibular ones, proprioceptive inputs are the most sensitive in detecting centre of pressure (COP) velocity^35^. Among the proprioceptors, muscle spindles, being stretch-sensitive and yielding information about the length and contraction of muscles^36^, are responsive to mechanical vibrations applied to muscle bellies or tendons.

When vibrations are applied, reflex responses arise (the tonic vibration reflex, TVR) and translate to an increased MU firing rate and a bigger electromyographic (EMG) response^37–39^. Conflicting studies are also present in the literature, including results suggesting that motorneuron excitability of spastic limbs is decreased after WBV, while that of the unaffected limb is immutated^40^. TVR seems to play a role even when vibrations are delivered to the whole body via an oscillating platform, as it occurs in whole body vibration (WBV) stimulation. WBV is in fact included in training and rehabilitative programmes as a mean to evoke neuromuscular responses from various muscles and therefore enhance muscle contractions^41^. Because TVR has been appointed as the main mechanism responsible for the enhanced sensitivity of muscle spindles primary endings^41–43^. WBV represent a reasonable candidate for the stimulation of proprioceptive structures. The goal of this study is to investigate if an appropriate use of mechanical vibrations-intended as a proprioceptive stimulation—can recalibrate the sensorimotor set employed to maintain balance in upright posture. Vibrations are delivered to specifically target muscles, soleus (SOL) and gastrocnemius lateralis (GL), that contribute the most to the primary strategy put into place during undisturbed human standing: the ankle strategy.

Our results confirm our hypothesis: postural control strategies are affected by vibratory stimulation. In particular, a greater interplay between the CNS and the periphery is observed in parallel to a recalibration of the sensorimotor set, appreciable not only as an observed increase of the cortical activity measured in beta band but also as a different set of muscle synergies employed to maintain balance.

## Results

To study the changes in balance control strategies, we recorded four one-minute trials of undisturbed balance before (baseline balance—BB) and after (post-stimulation balance—PSB) the delivery of WBV stimulation at 30 Hz that 17 participants received while on their fore-feet, which was shown to effectively target calf muscles^44^. Muscle activity was recorded from GL, SOL, tibialis anterior (TA), biceps femoris (BF) and rectus femoris (RF) of both legs; electroencephalographic (EEG) activity was recorded from 64 channels (10–20 arrangement), and centre of pressure (COP) trajectories via a force platform. All analyses were run both on the first minute and on all four trials, to evaluate eventual differences in the acute- and long-term response to the stimulation. Not all participants were able to reposition on the force platform within the given time, consequently the first trials after the WBVs were realigned and cropped appropriately across participants. This resulted in two subjects being removed from the acute-term analyses as the time occurred between the vibratory stimulations and the balance trials were too short and too long (11.6 s and 40.4 s). The alignment of balance recordings resulted in trials of the length of 44.8 s. The average time occurred between the WBV stimulation and the first balance trial was 27.9 ± 4.1 s. For the analyses on the longer-term effect, all 17 subjects were retained, and the concatenation of balance trials resulted in two 4-min long epochs.

### Dominant calf-muscles are targeted by the WBVs

To confirm that WBVs stimulated the targeted muscles (GL, SOL and TA), the root mean square (RMS) value of their EMG signals was analysed. Trials before and after the stimulation were compared to rule out any potential confounding factor given by motion artefacts during WBVs^45–48^. The median frequency (MF) of the EMG power spectrum was also analysed to exclude muscle fatigue^49^.

**Figure 1:**
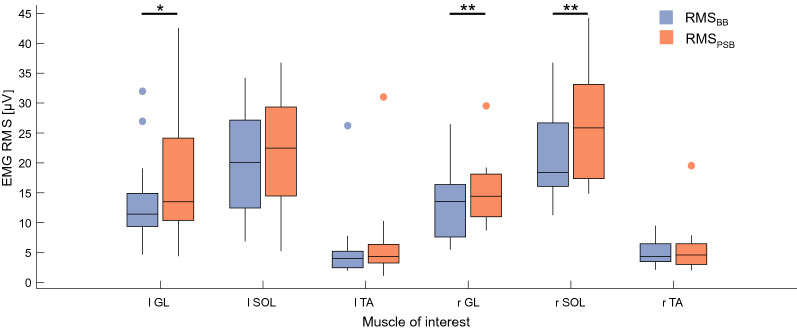
sEMG RMS before and after the WBVs. RMS values of lower limb muscles and their difference between the 45 s-long baseline (BB) and post-stimulation (PSB) trials: (*) and (**) indicate p < 0.05 and p < 0.01, respectively.

The acute-term analyses revealed that muscular activation increased especially among the muscles on the dominant side (the right for most subjects—11 out of 15): GL (*p* < 0.01) and SOL (*p* < 0.01) measured a bigger contraction after the vibrations were delivered. Similarly, a RMS_PSB_ bigger than RMS_BB_ was found also for the left GL (*p* < 0.05) (Fig. 1). The right GL and SOL –dominant muscles for 12 out of 17 subjects- showed an increased RMS_PSB_ even across the 4 min following the WBVs (*p* < 0.05). The only muscle that was found showing signs of fatigue was the left GL: its MF decreased significantly after the WBVs when computed over the 45 s-long sEMG signals and on the 4 min-long ones (*p* < 0.05). Otherwise, the power spectral density (PSD) of the other muscles did not shift towards lower frequencies after the stimulation.

### Acute-term increase of CNS-periphery interplay

**Figure 2:**
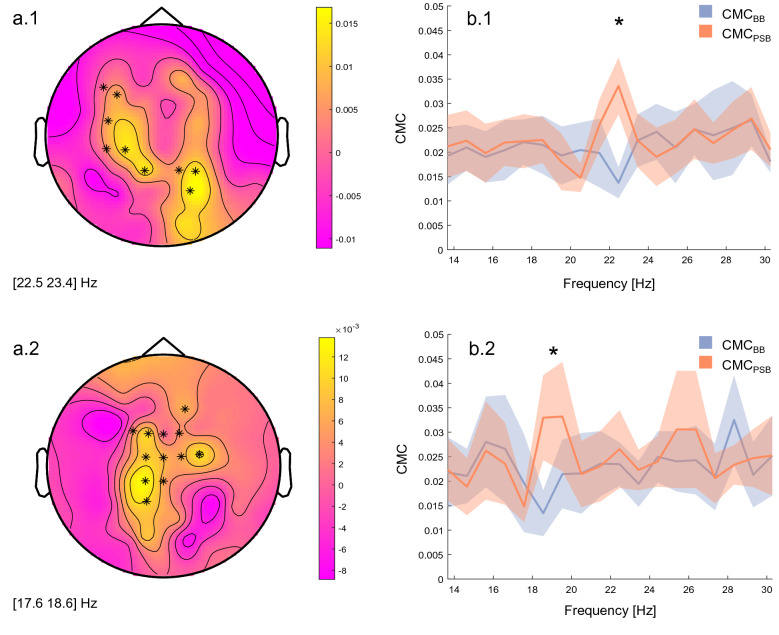
Corticomuscular coherence topographic maps and spectra. On the left-side, voltage topographic maps of the difference between EMG-EEG couplings before and EMG-EEG couplings after the WBVs (CMC_BB_ and CMC_PSB_, respectively), averaged across subjects, are reported for the right GL (**a.1**) and the right SOL (**a.2**). The asterisks depict electrodes that showed a significantly bigger CMC around 23 Hz (**a.1**) and 19 Hz (**a.2**). On the right-side, CMC spectra (mean ± standard error) of EMG-EEG couplings before and after the stimulation are reported for the right GL (**b.1**) and the right SOL (**b.2**). The asterisks depict the frequency bin at which the CMC signals differed between conditions, as identified by the cluster-based permutation test. Results are shown for CMC vectors obtained from the 45 s-long baseline (BB) and post-stimulation (PSB) trials.

Proprioceptors detect the muscle length changes during the natural back and forward body oscillation while standing still^35^. The information originating at these sensorial structures is then processed and integrated at higher levels to shape appropriate corrective postural responses^4^. Therefore, we hypothesised that, by stimulating these structures, we would have increased such exchange of information and this would have had a measurable impact on the balance control sub-systems. To investigate how the interplay between the CNS and the periphery was affected by WBVs, CMC was studied between those muscles that play the biggest role in actuating postural responses during undisturbed balance -the calf muscles (SOL, GL and TA)- and the EEG channels. The permutation test run on 45 s-long epochs detected two significant clusters in correspondence of a bigger CMC after the WBVs. Specifically, a stronger coupling was observed around 22 Hz between the right GL and electrodes situated mostly in the contralateral hemisphere (p < 0.05, Fig. 2a.1*–b.1). The effect had the same directionality for the right SOL and the same central contralateral electrodes but was centred around 18 Hz (p < 0.05, Fig. 2a.2–b.2). No significant difference was identified on the CMC vectors obtained from the concatenated EEG and sEMG epochs.

### Acute-term increase of cortical power in beta band

**Figure 3:**
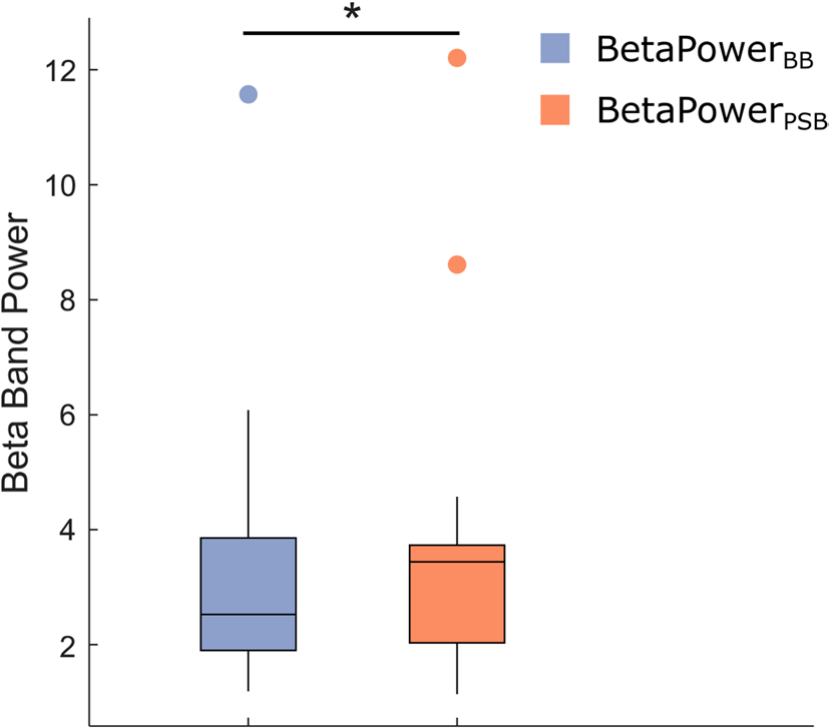
Changes in beta band activity. Box plot of the average power estimated in beta band (area under the curve, averaged over S1 channels) for EEG epochs recorded at C3, C4, CP3 and CP4 during balance trials before (purple) and after (orange) the vibratory stimulation. Significance (*p* < 0.01) is indicated by the asterisk and results are reported for 45-s long epochs.

Since the activity recorded in beta frequency range—mostly confined to S1—is suggested to facilitate proprioceptive feedback integration^33^, we expected a greater extent of information processing, observable as an increase of cortical activity in such frequency range, when stimulating proprioceptive structures via WBV. Further motivated by the higher CMC observed after the WBV, we used a one-side Wilcoxon test to test the hypothesis that even S1 beta-band activity would increase after the stimulation. BBA was quantified as the average power in beta band (15–30 Hz) recorded from sensors overlying S1^50^ and the test did indeed show that it increased in the acute-term balance after the WBVs (p < 0.01, Fig. 3), but not in the longer one.

### Acute-term recalibration of muscle networks

**Figure 4:**
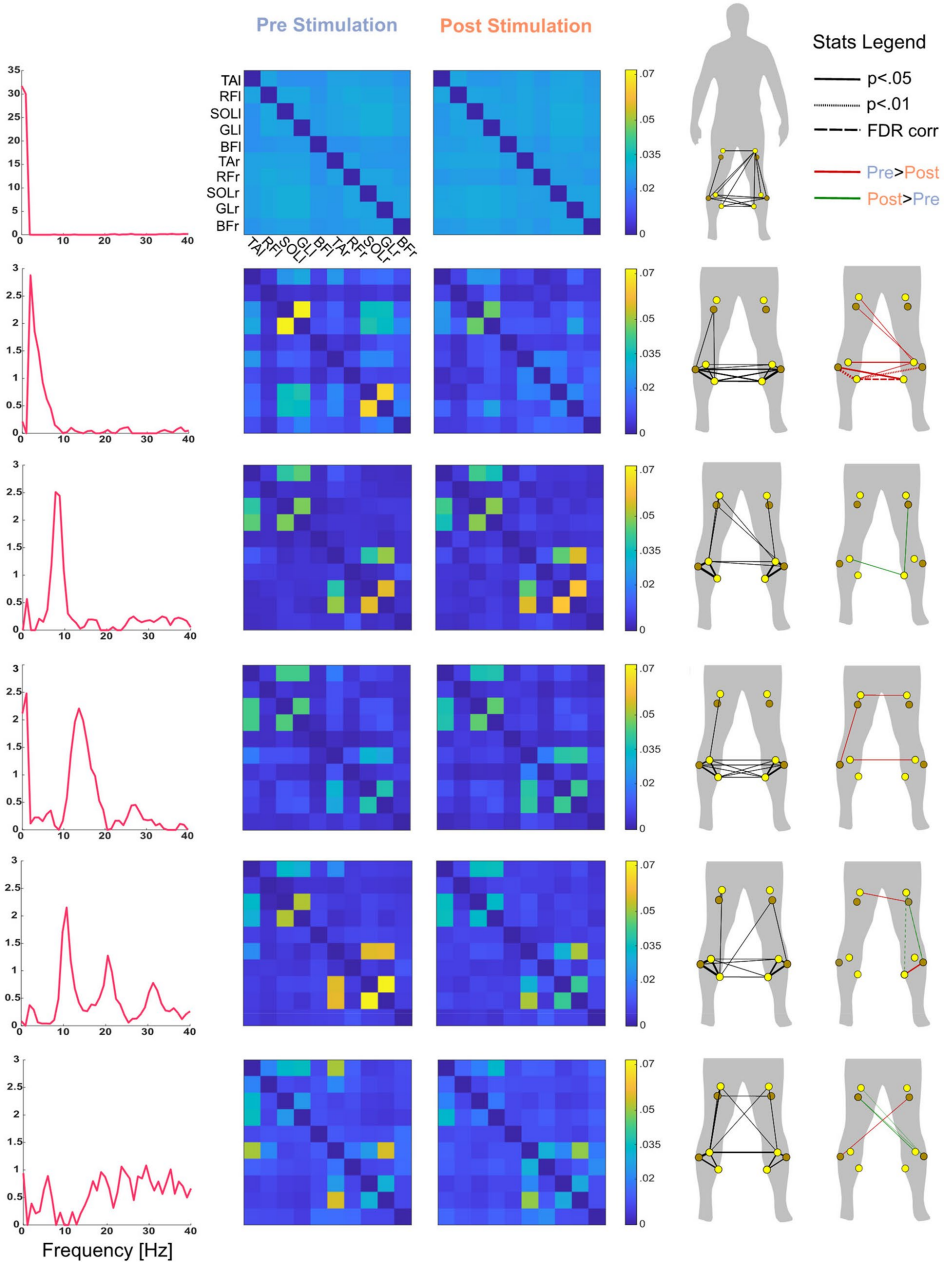
Edge-wise analysis of muscle networks. The left column depicts the six frequency components in which the coherence spectra were decomposed via NNMF (one for each row). The second and third columns show the adjacency matrixes, averaged across subjects, obtained for each frequency component from the 45-s long trials before and after the stimulation, respectively. These matrixes give the connection strengths, at different frequencies, between the 10 muscles (nodes of the network). The fourth column depicts the muscle networks averaged across subjects and conditions: the weight of the edges is obtained by averaging the two adjacency matrixes (pre and post stimulation) for each frequency component. The widths of the edges in column 4 reflect the strength of the connection. The fifth column depicts the statistical results of the edge-wise analysis. For each frequency component, only those edges that were significantly different (*p* < 0.05) between conditions (pre vs post) are depicted. Comparisons that resulted particularly significant (*p* < 0.01) are depicted via a dotted line; comparisons that resulted significant after FDR correction are depicted via a dashed line. The colours of the edges reflect the direction of the effect, with red lines indicating that the connection strength decreased after the stimulation and green lines indicating an increase after the stimulation. The width of green and red lines indicates the size of the effect.

IMC was computed between the 45 pairs of muscles (5 muscles per leg) as the coherence between the two EMG signals, both in the acute- and long-term, to investigate changes in the recruitment pattern of muscles during the balance task. The IMC spectra were factorised into a matrix of weights and a matrix of basis vectors. The latter yielded the six frequency bands over which the IMC was decomposed. Its elements were reorganised, for each frequency component, into two adjacency matrices (10 × 10) that reflected the coherence between each muscle pair before and after the WBVs^11^. IMC reflects the common neural input to two different muscles and therefore represents the extent to which the muscles are jointly recruited^7^. Network analyses were employed to investigate the level of changes induced by WBV stimulation. We quantified changes in the clustering coefficient (CC, reflecting network segregation) and participation coefficient (PC, reflecting network integration), as well as changes in the connection strength between the nodes of the networks (muscles). The six components obtained from the factorization of the coherence spectra of 45-s long epochs are displayed in Fig. 4. The first 4 components represent spectra peaking at specific frequencies: 0 Hz; 2 Hz; 8 Hz and 14 Hz. The fifth component is characterized by three frequency peaks at 10, 20 and 30 Hz. The last component instead does not show any predominant frequency. The edge-wise analysis yielded the following results (Fig. 4):

- The connection strength between the two SOL muscles decreased at 2 Hz after the stimulation (*p* < 0.05, FDR corrected). In addition, the connections between left GL-right SOL and between right SOL-right GL decreased after the WBVs (*p* < 0.01);
- The connection between left RF and left BF at 8 Hz increased after the stimulation (*p* < 0.05, FDR corrected);
- The strength of the connection between left RF and the left SOL increased after the WBVs (*p* < 0.05, FDR corrected), for the broad frequency component peaking at 10, 20 and 30 Hz;
- The connection strength between left GL-right RF and left GL-right BF increased after the WBVs (*p* < 0.01) in the broad frequency component.

**Figure 5:**
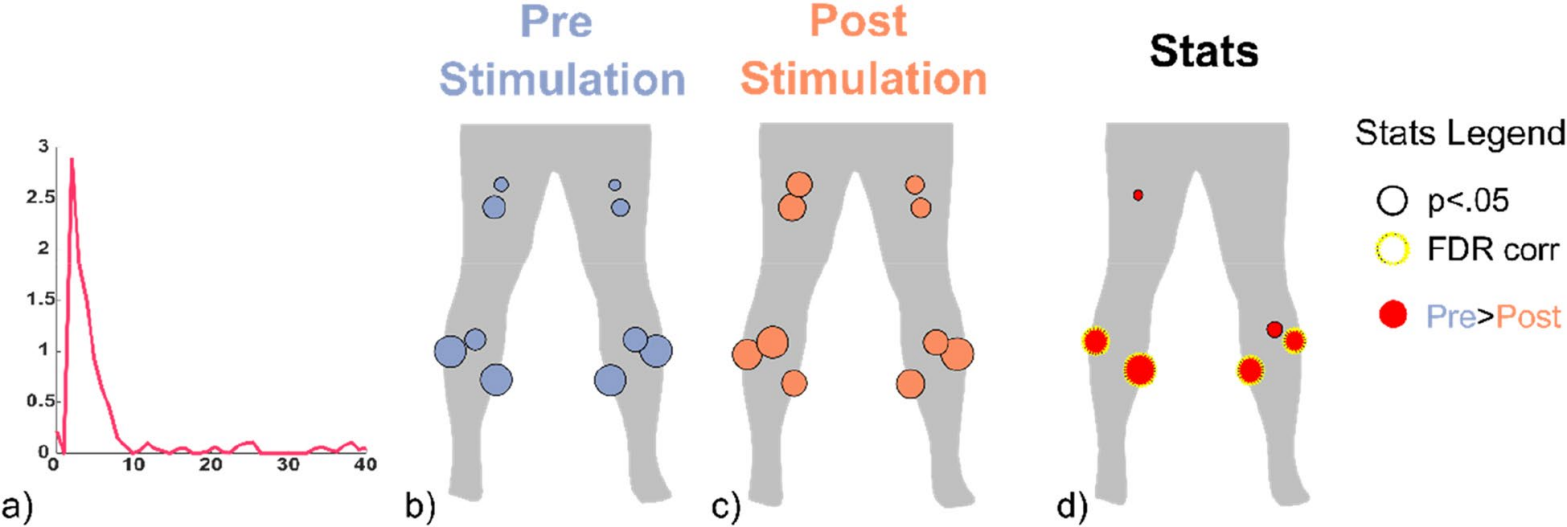
Node-wise analyses of muscle networks. Clustering coefficient results are reported for the frequency component where at least one node changed significantly between conditions. (**a**) Depicts the frequency component (2 Hz). (**b**,**c**) show the graph measure values for each node before and after the stimulation, respectively. (**d**) represents the statistical results of the node-wise analysis. The nodes that were significantly different (*p* < 0.05) between conditions (pre vs post) are depicted and those that survived FDR correction are contoured by a yellow line. The colours of the nodes reflect the direction of the effect, with red nodes indicating that the specific metric decreased after the stimulation. The size of the nodes indicates the size of the effect.

The node-wise analysis showed a significant decrease in segregation of the calf muscles at 2 Hz. Specifically, the CC of right SOL, left SOL, right GL and left GL decreased significantly after the stimulation (*p* < 0.05, FDR corrected) (Fig. 5).

Similar frequency components were obtained from the concatenated epochs. However, although a decrease of CC was measured at 2 Hz for both the left and right soleus, these results did not pass the FDR correction. Similarly, a decrease in the strength connection between left SOL-right SOL and between left SOL-left GL was significant, but did not pass the FDR correction.

### Acute-term instability is restored in the long-term

**Figure 6:**
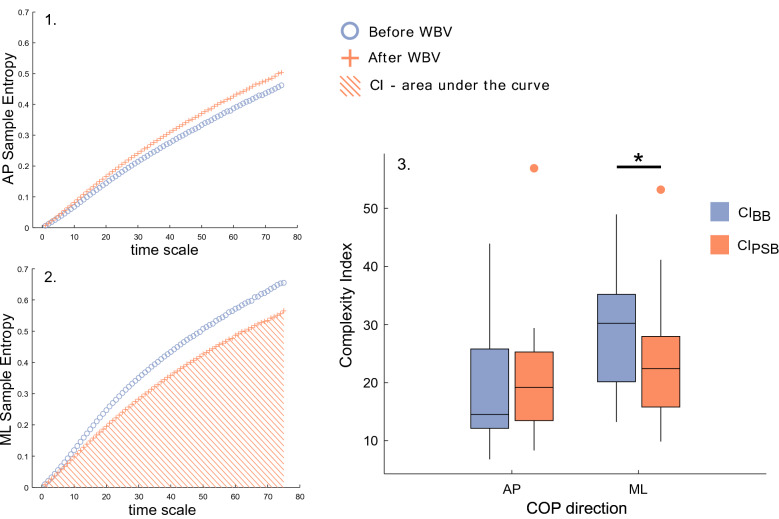
Multiscale sample entropy and complexity index. Plots of sample entropy averaged across subjects in the anterior–posterior (**1**) and in the medial–lateral direction (**2**) before and after the WBVS (round and cross marker, respectively). In (**2**), the area under the curve is displayed to represent the calculation of the CI for the ML direction, post stimulation. In (**3**) boxplots of the complexity index are displayed for the ML and AP direction (CI_ML_ and CI_AP_) and significant differences are depicted by the asterisk (*p* < 0.05).

Finally, to fully characterise the changes occurred in the postural mechanisms due to the WBVs, we analysed the COP trajectories in the anterior–posterior (AP) and medial–lateral (ML) directions. We extracted common metrics such as the mean distance (MD) and mean velocity (MV) values, and more novel ones, such as the complexity index (CI) that quantifies the level of regularity of the signal. An efficient postural control is the one that does not require particular attention and is reflected by a greater irregularity of the COP trajectories and therefore a greater CI^51,52^. Wilcoxon tests run on 45-s long trials revealed that MD_ML_, MV_ML_ and MV_AP_ increased immediately after the WBV (*p* < 0.001,* p* < 0.01,* p* < 0.001 respectively), while the mean distance covered by the COP in the anterior–posterior direction was not affected by the WBV stimulation (*p* > 0.025). Moreover, CI_ML_ decreased significantly in the minute following the mechanical vibrations (see Fig. 6). When the analyses were run on 4-min long trials, the postural control in the ML direction appeared recovered and the only significant results concerned metrics in the AP direction, registering a significant increase in MV_AP_ and CI_AP_ after the WBVs (*p* < 0.001 and *p* < 0.05 respectively).

## Discussions

While it is renowned that *during* WBVs muscle contraction is boosted, this is the first study that demonstrates that an increased muscle activation persists even *after* the stimulation, during upright standing. Although there is no universal consensus on whether WBV increases or decreases motorneuron excitability of healthy limbs^40,53^, this increased and sustained muscle activation—measured as a bigger EMG RMS across both GLs and the right SOL—is likely to be consequential to an enhancement in the sensitivity of the spindles of the related muscles^41^. As EMG RMS is not a direct measurement of muscle spindle activity, to confirm our results we also assessed if the increase was caused by muscle fatigue (median frequency shifting). Our data confirmed that the median frequency of the dominant GL and SOL did not change, indicating that their sEMG increase was not due to fatigue but rather reflecting a sustained proprioceptors sensitivity, maintained even once the stimulation was off.

This inference relates well to our results on corticomuscular coherence supporting the hypothesis of a greater information exchange between the muscles and the cortex occurring after the WBVs^16^. CMC has been historically utilised to describe the cortical drive to the muscles^12^, but recent findings support the idea that it also reflects afferent couplings from the periphery to the cortex itself^13^. We may therefore speculate that more sensitive muscle proprioceptors translated into a more abundant flux of proprioceptive feedbacks from the relevant muscles—right SOL and GL. However, considering also the original interpretation, the increased CMC measured after the WBVs points to an overall greater interplay between afferent and efferent signals that from the periphery reached the brain—and vice versa—with the purpose of detecting perturbations and correcting them via muscle modulation.

The enhanced activity observed in beta band after the stimulation supports the second and more recent interpretation. An increased BBA in the somatosensory areas of the brain—measured as a greater spectral power over C3, CP3, C4 and CP4—indicates a greater attempt of the sensorimotor system to preserve the status quo^33,54^. More specifically, a bigger BBA allows the system to more efficiently process proprioceptive signals from the periphery with the ultimate goal of *recalibrating* the sensorimotor set^33^ or, in this case, adjusting the COP position after an unexpected loss of balance.

The increased need for recalibration and the increased CMC observed after the WBVs are well justified by the fact that the ability of balancing declined immediately after the stimulation. In fact, we observe a clear degradation of stability in the medial–lateral direction after the mechanical stimulation, as shown by a larger COP displacement and velocity in the ML plane^55^. In addition to these unambiguous results, a reduced complexity of postural sway in the ML direction further confirms the reduction of the ability to maintain balance and the increased attempt of the system to maintain an upright stance. A smaller CI does in fact indicate a bigger regularity of COP paths, which is positively correlated to the attention needed to balance and reflects a less automatic postural control^51,52^. From our results it could therefore be inferred that, being the ML COP trajectories more regular after the WBVs, participants employed a greater amount of effort to maintain the same upright stance, as BBA results indicate too. The observed balance disruption in the ML direction is reasonable as this is the one that is mostly affected by the WBVs. In the AP direction instead, we observe only an increased COP velocity, which—if not associated with a bigger COP displacement—is not easily associated to worsening balance^55,56^.

To link the posturographic results with the electrophysiological ones, when the postural instability grows, lager COP movements lead to greater uncertainty in the periphery, which triggers a bigger demand for oscillatory recalibration^34^.

The increased need for cortical recalibration is reflected in the modified muscular networks that are observed after the WBVs (Figs. 4 and 5), where the weights of the networks are modified—or *recalibrated*—across different frequency ranges. Specifically, the greatest changes are observed in correspondence of those muscles from which the affluence of afferent information increased, namely the SOL and GL. Changes in connection strength are observable across these muscles—bilaterally—especially at very low frequencies (< 5 Hz), which resemble the frequency ranges reported by previous studies on IMC during balance^11,23^. Moreover, significant differences are detectable for the clustering coefficient at the very same frequency, where it decreases after the stimulation for the bilateral SOL and GL.

This could lead to the speculation—well aligned to the current literature (see next paragraph)—that these muscles were less synchronised with the rest of the network and therefore modulated more individually.

It is in fact suggested that IMC at frequencies below 6 Hz reflects subcortical inputs or reflexes, while IMC at higher frequencies reflects cortical ones^57^. In details, the first backs stiffness control while the second supports muscle control via synergy formation. In our case, the reduced CC found for the plantarflexors indicate a reduction of synchronisation of the latter with other neighbouring muscles—or reduced IMC—for frequencies below 5 Hz. This might suggest that subcortical inputs were less employed for the modulation of the plantarflexors after the WBVs. Because the plantarflexors were significantly more active after the stimulation and their corticomuscular coherence at higher frequencies increased but less spinal modulation was present, it is possible that more cortical control was employed for these muscles. This possible shift toward cortical control—observed during the more challenging task of *balancing after the stimulation*—is in line with recent findings suggesting that postural threat leads to a shift toward more supraspinal control of balance^58^. Different views are present in the literature in regard of whether IMC at low frequencies reflects modulation via subcortical and spinal inputs^59,60^, but besides the origin of such modulation, the network reconfiguration observed after the WBVs suggest that a *change in the modulation* of muscle activation has occurred after the stimulation while a cortical-muscular loop is likely to have played an important role in this *recalibration*.

As for the long-term analyses, significant results are found only for the sEMG and COP data. The first ones indicate that WBVs’ effect on the enhanced activation of the dominant calf muscle, differently than the recalibration effect, persisted for 4 min.The interpretation of posturography results is instead less straightforward. When computed across the four minutes after the WBVs, the COP parameters seem to stabilise in the ML plane and only an increase of COP velocity in the AP direction is found, which, if not associated with an increase of COP displacement, is more insidious to interpret^55^. Davids et al. did in fact report a smaller mean COP velocity in participants who had an anterior cruciate ligament complete rupture than in the control group^56^, contradicting the common belief that a higher velocity reflects a worse ability of controlling posture. The simultaneous recording of a significant increase of mean velocity and no significant change of COP mean distance might suggest that the average amount of time necessary to cover a fixed distance decreases after vibrations are applied. Although not straightforward, it is reasonable to infer that the decrease of time between one COP position and the next one might be linked to an increased muscle’s ability to adjust their length to counterbalance a body sway. This interpretation is supported by what the AP CI results suggest: that not only balance was restored in the long term, but also that was possibly improved, as the increased AP complexity index suggests^51^.

## Conclusions

Our results indicate that WBVs undermines balance in the first place, triggering the need for a bigger effort to control the upright stance and shifting muscle modulation toward supraspinal control, resulting in a recalibration of muscle recruitment. However, the system seems to recover from such disruption and regain control over a longer time interval. Indeed, while muscle recruitment and cortical effort appear unaltered over the long term, the balance seems not only restored but also improved, besides the still clearly affected calf muscles. Our work suggests that *WBV stimulation is worth further investigations as a mean to recalibrate postural control mechanisms and potentially restore balance.* Moreover, our results provide new perspectives on WBV applications, paving the way for future research on the interaction between the CNS and the peripheral muscles.

## Materials and methods

### Subjects and experimental design

Eleven females and six males (age: 23.06 ± 2.51 years; height: 167.22 ± 9.74 cm; mass: 61.32 ± 10.69 kg) volunteered in the study after giving written consent. History of neuromuscular or balance disorder and recent injuries to the lower limbs were the exclusion criteria. Among the inclusion criteria, participants were required to do more than 5 h per week of physical activity to ensure a minimum of athleticism. Even though WBV is a mild vibratory stimulation, the 4-min-long postural task requires a certain level of muscular engagement. Participants were also asked to not consume alcoholic beverages and to not assume medication over the 24 h prior to the experiment. The protocol of the study received approval by the Ethics Committee on Life and Health Sciences of Aston University.

A single-group, repeated-measure design was used and the data were collected at the Aston Laboratory for Immersive Virtual Environments. Electroencephalographic (EEG), COP and surface EMG (sEMG) signals of calf muscles were collected while participants underwent a balance task, performed before and after WBV mechanical stimulation.

### Experimental protocol and data recording

A familiarisation session was run for participants to get acquainted with the WBV device before the study began. After the recording equipment was set up, the first part of the study consisted in recording four baseline balance (BB) trials of 60 s. Participants were instructed to “stand as still as possible”^61^ with feet shoulder-width apart and arm along the trunk while fixating their gaze at a tape cross placed on the wall in front of them, at approximately 2.5 m distance. After the baseline trials were collected, the second part of the study took place: a one-minute vibratory stimulation was delivered with a side-alternating platform (Galileo^®^ Med, Novotec GmbH, Pforzheim, Germany) that operated at 30 Hz, peak-to-peak amplitude of 4 mm. While undergoing the WBVs, participants stood on their forefeet, knees unlocked, keeping contact between heels and a 4 cm tall foam parallelepiped glued to the platform^62^. This combination of WBV settings (stimulation frequency and subjects’ posture) was chosen as it triggers the greatest response of the plantarflexors muscles—SOL and GL^44^. The WBV stimulation was followed by the recording of four balance trials of 60 s each, which will be referred to as Post-Stimulation balance (PSB) trials. After the WBVs, participants were asked to position themselves on the platform as soon as they felt ready to undergo the postural task. We will refer to BB and PSB as the two conditions tested in this study.

EEG and sEMG data were acquired using EEGO sports ES-232 (ANT neuro, Enschede Netherlands). To collect the electrical brain activity (EEG), a 64-channel Ag/AgCl wet-electrode waveguard cap was used in connection to a portable amplifier fixated on the participants’ backs. Data were continuously collected with a standardised 10–20 system montage and were sampled at 1000 Hz. Caps of different sizes were fitted on the head of participants; the correct placement was obtained by checking that Cz was placed at mid-distance between the nasion and inion anatomical points and at mid-distance between the left and right lobes. Muscle signals (sEMG) were collected via bipolar Ag/AgCl electrodes (Arbo Solid Gel, KendallTM, CovidienTM 30 mm × 24 mm, centre-to-centre distance 24 mm) that were connected to the portable amplifier via cascaded bipolar adaptors XS-271.A, XS270.B, XS-270.C. Electrodes were placed over the TA, GL, SOL, RF and BF muscles of both legs and arranged along the presumed direction of muscle fibres, as recommended by the SENIAM guidelines^63,64^. The reference electrode was placed over the tuberosity of the right tibia. To reduce inter-electrode resistance, the skin area was shaved and degreased by mean of light abrasion with a disinfectant. To quantify balance, COP trajectories were recorded and sampled at 1000 Hz via an AMTI OR 6–7 force platform (Advanced Mechanical Technology, Watertown, MA, USA) connected to a motion capture system (Vicon Nexus, Vicon Motion Systems Limited, Oxford, UK). A LabJack U3-HV acquisition unit was programmed in Matlab^®^R2019a (The Mathworks, Inc., Natick, MA) with a custom-made script to synchronise electrophysiological acquisitions and posturography data. The trigger signal was sent as a 5 V TTL signal to the DB25 port of the EEGO sports master amplifier and as a 1.25 V TTL signal to the rear of the Vicon Lock unit.

### Data synchronisation

Since participants reacted differently to the stimulation, different delays were recorded between the WBV stimulation and the first PSB trial.

To allow a consistent comparison while evaluating the *acute* effect of WBV on balance, the first BB and PSB trials were preprocessed to match the delay between the WBVs and the first PSB across participants. The distribution of the time employed by each participant to reposition on the force platform (delays) was analysed and the subjects whose delay represented an outlier values were removed from the dataset. The recordings (EEG, sEMG and COP) of the retained subjects were then synchronised to those of the subject that scored the greatest delay and were further cropped to match the duration of the shortest recording. The same cropping procedure was applied to the first BB trials, which were cropped at the same time point of the corresponding PSB trial to make the trials collected before and after the WBVs comparable. These preprocessed signals will be hereafter referred to as the *cropped* ones.

The *long-term* effect of WBV stimulation on balance was evaluated by concatenating the four BB trials and the four PSB trials into two 240 s-long epochs, which were then used for analyses. These signals will be hereafter referred to as the *concatenated* ones.

### Data preprocessing: EEG and sEMG

The cropped and the concatenated EEG epochs were preprocessed in Matlab^®^R2019a (The Mathworks, Inc., Natick, MA) in a semi-automatic fashion, following the steps proposed in the software Cartool^65^. The EEG signals were de-trended, mirrored to avoid edge-artefacts, filtered in beta band (13–32 Hz) with a zero-phase FIR filter, cropped and de-trended again. Eye-blinks were removed automatically by subtracting the signals recorded at FP1, FPz and FP2 sites from the remaining 60 EEG channel data, proportionally to their contribution to each channel^66,67^. Channels with standard deviation (SD) exceeding the average SD by a factor of 1.7 were replaced by the average of the signals from neighbouring channels^10^. The value of the factor was placed at 1.7 after a visual inspection—confirmed also by a second researcher—of the preprocessed channels confirmed that this threshold was not too conservative, leading to the interpolation of channels exceeding the physiological range of ± 80 μV^10^. Channels were finally re-referenced to the common average.

The cropped and the concatenated sEMG epochs were preprocessed and analysed in Matlab^®^R2019a (The Mathworks, Inc., Natick, MA), using custom-made scripts. The power line noise was occasionally present not only at 50 Hz, but also at superior and inferior harmonics. To suppress such noise, a comb stop-band filter was used at harmonics between 25 and 150 Hz, using a stop band of 1 Hz. The signals were band-pass filtered between 20 and 260 Hz with a zero-phase Butterworth filter, after padding was applied to avoid edge-artefacts. Filtered sEMG epochs were then rectified^12,68^ with a Hilbert transform, which provides results similar to a full-wave rectification^28,68,69^. The preprocessing was done using Fieldtrip ft_preprocessing function^70^.

### Data analysis

#### CMC

CMC analyses were performed only on those muscles targeted by the vibrations: the lower limb ones (SOL, GL and TA). Coherence estimates were computed between the preprocessed EEG and sEMG signals (both between the cropped epochs and the concatenated ones), for a total of 360 couplings [EEG channels (60) × muscles (6)] per condition. Power spectral densities (PSD) of EEG and sEMG signals were estimated with Welch’s averaged periodogram method, segmenting the epochs in Hamming windows of one seconds and zero overlap^30^. The CMC between the signal $x$—EEG and $y$—sEMG was then calculated as:

$${C}_{xy}\left(f\right)=\frac{{\left|{P}_{xy}\left(f\right)\right|}^{2}}{{P}_{xx}\left(f\right){P}_{yy}\left(f\right)},$$

where ${P}_{xy}$ is the cross-spectral density between the input signals for a given frequency $f$, and ${P}_{xx}$ and ${P}_{yy}$ are the auto-spectral densities of $x$ and $y$, respectively. In total, 720 CMC vectors [EEG channels (60) × muscles (6) × condition (2)] were retained for statistical analyses.

#### BBA

To quantify beta band activity changes between before and after the WBVs, the beta power-defined as the average area under the PSD curve in [15–30 Hz]—was used as a summary statistic^17^. PSDs were estimated with Welch’s averaged periodogram, using non-overlapping Hamming windows of one second, and were obtained for cropped and concatenated EEG epochs recorded from the sensors overlying the sensorimotor cortex (C3, C4, CP3 and CP4)^50^. For each subject, the area under the curve in beta frequency range was averaged across the four channels and the two resulting values (${BetaPower}_{BB}$ and ${BetaPower}_{PSB}$) were retained for statistical analyses.

#### IMC and muscle networks

sEMG signals from *all muscles* were instead used for connectivity analyses. IMC was estimated between all muscle pairs using *mschoere* Matlab function. Forty-five combinations (${C}_{n,k}$) resulted from the formula:

$${C}_{n,k}=\frac{n!}{k!\left(n-k\right)!},$$

where $n=10$ is the total number of muscles (nodes) and $k=2$ is the number of muscles in each arrangement (pair). Magnitude-squared coherence values were computed over sEMG power spectral density (PSD) using Welch method, with window length of 1 s and overlap of 75%^11^. To break down the connectivity values into different frequency components and to make the coupling strength comparable between conditions, a non-negative matrix factorization (NNMF) algorithm was run on a $N\times [M\times P\times C]$ (frequency bin × [muscle pairs × participants × conditions]) weighted undirected connectivity matrix. *Nnmf* Matlab function was used to decompose the coherence spectra (0–40 Hz) in two matrixes ${W}_{AllSubj}$ $[N\times K]$) and ${H}_{AllSubj}$ $[K\times [M\times P\times C]]$, where $K$ equals 6 and is the number of frequency components in which the matrix was factorised. 5000 iterations were used. The two matrixes were then reshaped into subject-specific $W$ $[N\times K]$ and $H$ $[K\times M]$ for both conditions (${W}_{BB} [42\times 6]$ and ${H}_{BB}[6\times 45]$; ${W}_{PSB} [42\times 6]$ and ${H}_{PSB}[6\times 45]$). For every subject, $H$ was reshaped into six weighted undirected connectivity matrixes $C$, which yielded the connection strengths between nodes (muscles) in every frequency band. C_BB_ and C_PSB_ resulted in $10\times 10$ connectivity matrixes. To conduct a static node-wise analysis, two graph measures were calculated for every node of the subject-specific C_BB_ and C_PSB_ of every frequency component: clustering coefficient (CC, reflecting segragation) and participation coefficient (PC, reflecting integration)^71^. Themeasures obtained from the same muscle were used for statistical analysis. To run a static edge-wise analysis, the connection strengths of the 45 muscle pairs yielded by C_BB_–C_PSB_ were retained for statistical analysis.

#### sEMG

A root mean square (RMS) value was obtained from the preprocessed sEMG epochs of those muscles targeted by the WBVs (SOL, GL and TA) before and after the stimulation. In total, 12 [muscle (6) × condition (2)] RMS values were obtained for each participant and retained for statistical analysis. To quantify the level of muscle fatigue induced by the stimulation, a frequency parameter was extracted from the filtered unrectified sEMG epochs. Specifically, the median frequency (MF_BB_–MF_PSB_) of the sEMG power spectrum—computed with the same specifics used for IMC estimation—was identified for each participant and for each muscle as the frequency that divides the spectrum in two parts of equal power^49^.

#### COP

The cropped COP coordinates and the concatenated ones were analysed in Matlab^®^R2019a (The Mathworks, Inc., Natick, MA) with custom-made scripts. The mean of each signal was subtracted from anterior–posterior (AP) and medial–lateral (ML) COP coordinates, which were then low-pass filtered at 12.5 Hz using a 4th order Butterworth filter^51^. For each condition and each participant, as described in Ref.^72^, COP mean distance (MD) and COP mean velocity (MV) values were computed for both directions: MD_AP_, MD_ML_, MV_AP_ and MV_ML_. The four stabilometric parameters were normalised by participant’s height, weight and age by applying a simultaneous detrending normalisation^73^.

To compute the multiscale sample entropy (MSE), the preprocessed AP and ML time-series were further normalised by their standard deviation values^51^. MSE was obtained with a Matlab File Exchange function and the recommended default values of the pattern length and similarity criterion were used^74,75^. The complexity index (CI) was computed as the sum of the individual sample entropies obtained for every time scale in both directions—ML and AP—obtaining CI_ML_ and CI_AP_ respectively^52,76^. For the long-term analyses—due to the dimension of the data—the MSE was not computed on the concatenated epochs, but on the individual epochs of one minute. The MSE values obtained from the four epochs preceding (and following) the WBVs were averaged to obtain ${MSE}_{BB}$ (and ${MSE}_{PSB}$) that were used to compute the respective complexity index values.

### Statistical analysis

#### CMC

For every muscle, the 60 CMC vectors obtained from BB trials were compared to the respective ones obtained from the PSB trials via mean of a cluster-based permutation test^77^, which was carried out in Fieldtrip using 2000 permutations^70^. Comparisons were run in the beta frequency range and this procedure was applied to the CMC vectors obtained from both the cropped and concatenated EEG and sEMG epochs.

#### BBA

A one-tailed Wilcoxon test was run to evaluate whether any significant difference was present in BBA in the somatosensory cortex between before and after the vibratory stimulation. The directionality of results from the permutation test was used to infer on the effect of WBVs on BBA.

#### IMC

Since we had no prior information on the effect of WBVs on muscular connectivity during balance, two-tailed Wilcoxon signed rank tests were used to test it. Tests were run for every muscle in every frequency component between the connectivity metric obtained from C_BB_ and C_PSB_. For the node-wise analysis, 4 × 10 × 6 ([metrics × muscles × frequency components]) tests were performed on the connectivity metrics obtained from the balance results. For the edge-wise analysis, 45 × 6 ([muscle pairs × frequency components]) tests were run on the connection strengths computed from the balance trials, respectively. To correct for the multiple comparisons, the False Discovery Rate (FDR)^78^ procedure was applied for every metric (CC, PC, BC, STR, edge) across muscles, within each frequency band.

#### sEMG

To quantify the differences in muscle activations between the balance trials (before and after WBV stimulation), six two-tailed Wilcoxon signed rank test were run between RMS_BB_ and RMS_PSB_, one for each muscle. To test the hypothesis that a greater level of muscle fatigue was induced by the WBVs, six one-tailed Wilcoxon signed tests were run between MF_BB_ and MF_PSB_, one for each muscle.

#### COP

Since we had no a priori expectation on the effect of WBVs on postural stability, a two-tailed Wilcoxon signed rank test was run for every normalised parameter (MD_AP_, MD_ML_, MV_AP_ and MV_ML_). Since two parameters were obtained from each time-series (ML and AP), a Bonferroni correction was applied to the significance level that was therefore set at 0.025. A Spearman correlation coefficient was used to quantify the level of collinearity between the COP parameters and the anthropometrics.

Another two-tailed Wilcoxon signed rank test was used to test for significant differences between complexity indexes measured in moth directions before and after the mechanical vibrations.

### Ethics approval

The study was carried out according to the Declaration of Helsinki (2013) and was approved by the University Research Ethics Committee at Aston University (reference number: 1561).

### Consent to participate

All participants provided informed consent before participating.

## Data Availability

Since sharing data in an open-access repository was not included in our participant’s consent and therefore compromises our ethical standards, data are only available on request from the corresponding author.

## Code availability

The code used for the analyses of the data will be shared upon request to the corresponding author.
